# Transoral surgery for superficial head and neck cancer: National Multi‐Center Survey in Japan

**DOI:** 10.1002/cam4.3927

**Published:** 2021-05-15

**Authors:** Chikatoshi Katada, Manabu Muto, Satoshi Fujii, Tetsuji Yokoyama, Tomonori Yano, Akihito Watanabe, Toshiro Iizuka, Shigetaka Yoshinaga, Ichiro Tateya, Hiroki Mitani, Yuichi Shimizu, Akiko Takahashi, Tomoyuki Kamijo, Noboru Hanaoka, Makoto Abe, Akihiro Shiotani, Koichi Kano, Yukinori Asada, Tamotsu Matsuhashi, Hirohito Umeno, Kenji Okami, Kenichi Goda, Shinichiro Hori, Yoichiro Ono, Shuji Terai, Yasuaki Nagami, Kenichi Takemura, Kenro Kawada, Mizuo Ando, Naoto Shimeno, Akihito Arai, Yasutoshi Sakamoto, Masaaki Ichinoe, Tetsuo Nemoto, Masahiro Fujita, Hidenobu Watanabe, Tadakazu Shimoda, Atsushi Ochiai, Takakuni Kato, Ryuichi Hayashi

**Affiliations:** ^1^ Department of Gastroenterology Kitasato University School of Medicine Sagamihara Japan; ^2^ Department of Therapeutic Oncology Kyoto University Graduate School of Medicine Kyoto Japan; ^3^ Department of Molecular Pathology Yokohama City University School of Medicine Yokohama Japan; ^4^ Department of Health Promotion National Institute of Public Health Wako Japan; ^5^ Department of Gastroenterology and Endoscopy National Cancer Center Hospital East Kashiwa Japan; ^6^ Department of Otolaryngology Keiyukai Sapporo Hospital Sapporo Japan; ^7^ Department of Gastroenterology Toranomon Hospital Tokyo Japan; ^8^ Endoscopy Division National Cancer Center Hospital Tokyo Japan; ^9^ Department of Otolaryngology ‐ Head and Neck Surgery Kyoto University Kyoto Japan; ^10^ Department of Head and Neck Oncology Cancer Institute Hospital Tokyo Japan; ^11^ Division of Endoscopy Hokkaido University Hospital Sapporo Japan; ^12^ Department of Endoscopy Saku Central Hospital Advanced Care Center Saku Japan; ^13^ Division of Head and Neck Surgery Shizuoka Cancer Center Nagaizumi Japan; ^14^ Department of Gastrointestinal Oncology Osaka International Cancer Institute Osaka Japan; ^15^ Department of Gastroenterology and Hepatology Okayama University Graduate School Okayama Japan; ^16^ Department of Otolaryngology‐Head and Neck Surgery National Defense Medical College Tokorozawa Japan; ^17^ Department of Otorhinolaryngology ‐ Head and Neck Surgery Kitasato University School of Medicine Sagamihara Japan; ^18^ Department of Head and Neck Surgery Miyagi Cancer Center Natori Japan; ^19^ Department of Gastroenterology Akita University School of Medicine Akita Japan; ^20^ Department of Otolaryngology‐ Head and Neck Surgery Kurume University School of Medicine Kurume Japan; ^21^ Department of Otolaryngology‐ Head and Neck Surgery Tokai University Isehara Japan; ^22^ Department of Endoscopy The Jikei University School of Medicine Tokyo Japan; ^23^ Department of Endoscopy NHO Shikoku Cancer Center Matsuyama Japan; ^24^ Department of Gastroenterology Fukuoka University Chikushi Hospital Chikushino Japan; ^25^ Division of Gastroenterology and Hepatology Graduate School of Medical and Dental Sciences Niigata University Niigata Japan; ^26^ Department of Gastroenterology Osaka City University Graduate School of Medicine Osaka Japan; ^27^ Department of Gastroenterology Ishikawa Prefectural Central Hospital Kanazawa Japan; ^28^ Department of Gastrointestinal Surgery Tokyo Medical and Dental University Tokyo Japan; ^29^ Department of Otolaryngology ‐ Head and Neck Surgery Graduate School of Medicine University of Tokyo Tokyo Japan; ^30^ Department of Gastroenterology Kobe City Medical Center General Hospital Kobe Japan; ^31^ Department of Otolaryngology‐Head and Neck Surgery Kyoto Prefectural University of Medicine Kyoto Japan; ^32^ Kitasato Clinical Research Center Kitasato University School of Medicine Sagamihara Japan; ^33^ Department of Pathology Kitasato University School of Medicine Sagamihara Japan; ^34^ Department of Diagnostic Pathology Showa University School of Medicine Yokohama Northern Hospital Yokohama Japan; ^35^ Department of Clinical Pathology Sapporo Hokuyu Hospital Sapporo Japan; ^36^ Department of Pathology Pathology and Cytology Laboratories BML INC Tokyo Japan; ^37^ Department of Diagnostic Pathology Shizuoka Cancer Center Nagaizumi Japan; ^38^ Exploratory Oncology Research and Clinical Trial Center National Cancer Center Kashiwa Japan; ^39^ Department of Otorhinolaryngology ‐ Head and Neck Surgery The Jikei University School of Medicine Tokyo Japan; ^40^ Department of Head and Neck Surgery National Cancer Center Hospital East Kashiwa Japan

**Keywords:** head and neck cancer, larynx preservation, pharyngeal cancer, superficial cancer, transoral surgery

## Abstract

Head and neck cancers, especially in hypopharynx and oropharynx, are often detected at advanced stage with poor prognosis. Narrow band imaging enables detection of superficial cancers and transoral surgery is performed with curative intent. However, pathological evaluation and real‐world safety and clinical outcomes have not been clearly understood. The aim of this nationwide multicenter study was to investigate the safety and efficacy of transoral surgery for superficial head and neck cancer. We collected the patients with superficial head and neck squamous cell carcinoma who were treated by transoral surgery from 27 hospitals in Japan. Central pathology review was undertaken on all of the resected specimens. The primary objective was effectiveness of transoral surgery, and the secondary objective was safety including incidence and severity of adverse events. Among the 568 patients, a total of 662 lesions were primarily treated by 575 sessions of transoral surgery. The median tumor diameter was 12 mm (range 1–75) endoscopically. Among the lesions, 57.4% were diagnosed as squamous cell carcinoma *in situ*. The median procedure time was 48 minutes (range 2–357). Adverse events occurred in 12.7%. Life‐threatening complications occurred in 0.5%, but there were no treatment‐related deaths. During a median follow‐up period of 46.1 months (range 1–113), the 3‐year overall survival rate, relapse‐free survival rate, cause‐specific survival rate, and larynx‐preservation survival rate were 88.1%, 84.4%, 99.6%, and 87.5%, respectively. Transoral surgery for superficial head and neck cancer offers effective minimally invasive treatment.

**Clinical trials registry number:** UMIN000008276.

## Graphical abstract

This is the first report on nationwide survival data and pathological criteria of superficial head and neck cancer. Transoral surgery for superficial head and neck cancer was safe and offered highly curative minimally invasive treatment preserving organs and their function. Subepithelial invasion was clinically useful predictor of recurrence after transoral surgery.

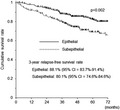

## 1 INTRODUCTION

Worldwide incidence and mortality of oropharyngeal cancer are reported as 92,887 and 51,005 and those of hypopharyngeal cancer are 80,608 and 34,984 in 2018.^1^ The prognosis is still poor even with multimodal treatment because most patients have locally advanced disease with lymph node involvement at the time of diagnosis and have a propensity for developing distant metastasis.^2^, ^3^

The standard treatment for resectable oro‐ and hypopharyngeal cancer is laryngopharyngectomy with pharyngeal reconstruction, leading to a loss of natural speech and a difficulty of swallowing.^4^ An alternative treatment is chemoradiotherapy, which can preserve organ and function. However, it often caused serious adverse effects, such as dysphagia, due to severe mucositis and xerostomia, negatively affecting patients’ quality of life.^5^

The ideal approach to improve the patients’ survival and to preserve organ and function is early detection of cancer and applying minimally invasive treatment.^6^ Tumor located within the epithelium and subepithelial layer was categorized as superficial cancer.^7^ Muto et al. reported that narrow band imaging (NBI: Olympus Co., Ltd.) enabled virtual chromoendoscopy and early detection of superficial head and neck cancer.^8^ Then, NBI is now widely used in clinical practice in many countries.^9^, ^10^, ^11^, ^12^, ^13^, ^14^, ^15^

For superficial lesions, endoscopic mucosal resection (EMR) and endoscopic submucosal dissection (ESD) have been indicated and showed effectiveness.^16^, ^17^, ^18^, ^19^, ^20^, ^21^, ^22^, ^23^, ^24^ Recently, transoral video‐assisted surgery (TOVS) and endoscopic laryngopharyngeal surgery (ELPS) have also been indicated.^25^, ^26^, ^27^, ^28^, ^29^ Together, EMR, ESD, TOVS, and ELPS are classified as transoral surgery (TOS). While TOS has been widely indicated for superficial head and neck cancer, their pathological evaluation is not standardized. Then, the clinical management after TOS is not also standardized. In addition, the real‐world effectiveness and safety of TOS for superficial head and neck cancer have not been well defined. We, therefore, conducted a national multi‐center survey of TOS in Japan.

## 2 MATERIALS AND METHODS

### 2.1 Participants

Patients who were primarily treated by TOS from April 2001 through July 2012 were retrospectively collected from 27 hospitals in Japan. The inclusion criteria were as follows: (a) tumors pathologically diagnosed as squamous cell carcinoma (SCC), (b) tumors invasion was pathologically limited within subepithelial layer, (c) no exposure of tumor cells to the vertical margin (negative vertical margin), (d) macroscopic tumor location in the oropharynx, hypopharynx, or supraglottis, (e) no regional lymph node metastasis on computed tomography, and (f) no other active advanced cancer in the head or neck region.

Written informed consent was obtained from all patients for the procedures in this study. Patients with concomitant primary cancer in any other organ were excluded. If cancers in other organs have been curatively treated when initial TOS was indicated, the patients were included. This study was approved by ethics committees in all participating hospitals and was registered in UMIN Clinical Trials Registry (UMIN000008276).

### 2.2 Transoral surgery (TOS)

TOS is defined as a procedure or an operation to perform mucosectomy for which a surgical device and visual guidance are inserted from mouth. Ablation procedure is not included in TOS. EMR and ESD were mainly performed by gastroenterologists. Others were mainly performed by head and neck surgeons.

### 2.3 Outcomes/Survey variables

The primary objective was effectiveness of TOS, and the secondary objective was safety including incidence and severity of adverse events. The survey variables were as follows: (a) the clinicopathological characteristics of patients with superficial SCC of head and neck, (b) adverse events associated with TOS, (c) incidences of local recurrence, regional lymph node recurrence, and distant recurrence after TOS and subsequent treatments, (d) incidence of and treatment regimen for metachronous cancer, and (e) the survival data on follow‐up duration (overall survival, relapse‐free survival, cause‐specific survival, and larynx‐preservation survival after TOS).

### 2.4 Histopathological analysis

One certified pathologist (S.F.) performed centralized pathology review of registered patients and excluded the patients without SCC, those with SCC with muscularis propria invasion, and those with histologic cancer types other than SCC. As a second step, 10 certified pathologists developed a new set of diagnostic criteria to distinguish subepithelial invasive SCC from SCC *in situ* for this study. The criteria were as follows; at least one solitary nest of epithelial neoplastic cells is present in the stroma clearly separated from intraepithelial carcinoma or intraepithelial carcinoma with a thickness of 500 μm or greater. As a third step, we conducted a central pathological review board by three certified pathologists (M.F., T.N., and M.I.). This board defined the presence or absence of invasion blinded to the clinical findings according to the diagnostic criteria. Consensus decision making was used to make final pathological diagnosis.

### 2.5 Local, regional lymph node and distant recurrence

Local recurrence was defined as a development of tumor at the treatment site of TOS. Regional lymph node and distant recurrence were defined as an abnormal enlargement of lymph node and a new lesion in distant location detected on the computed tomography, respectively.

### 2.6 Metachronous cancer

Metachronous cancer was defined as cancer detected in the region after initial TOS that was clearly separate from the resection scar. Metachronous cancer in other organs was defined as cancer arising in organs other than the head and neck after initial TOS.

### 2.7 Statistical analysis

*p*‐values for categorical data were calculated by using Kruskal‐Wallis test for trends in the median procedure times and using Fisher's exact test for other variables related to the safety of transoral surgery, respectively. Overall survival rates, relapse‐free survival rates, and cause‐specific survival rates were estimated using the Kaplan–Meier method and tested by log‐rank tests. Cumulative incidence of metachronous head and neck cancers, metachronous cancers arising in other organs, and larynx‐preservation survival that events were laryngectomy and all death were estimated using the Kaplan–Meier method. We defined the time to the development of a metachronous cancer as the period from the day of TOS to the day of diagnosis of a metachronous cancer. All data were analyzed with SAS (version 9). All authors had access to the study data and have reviewed and approved the final manuscript.

## 3 RESULTS

### 3.1 Participants

**FIGURE 1:**
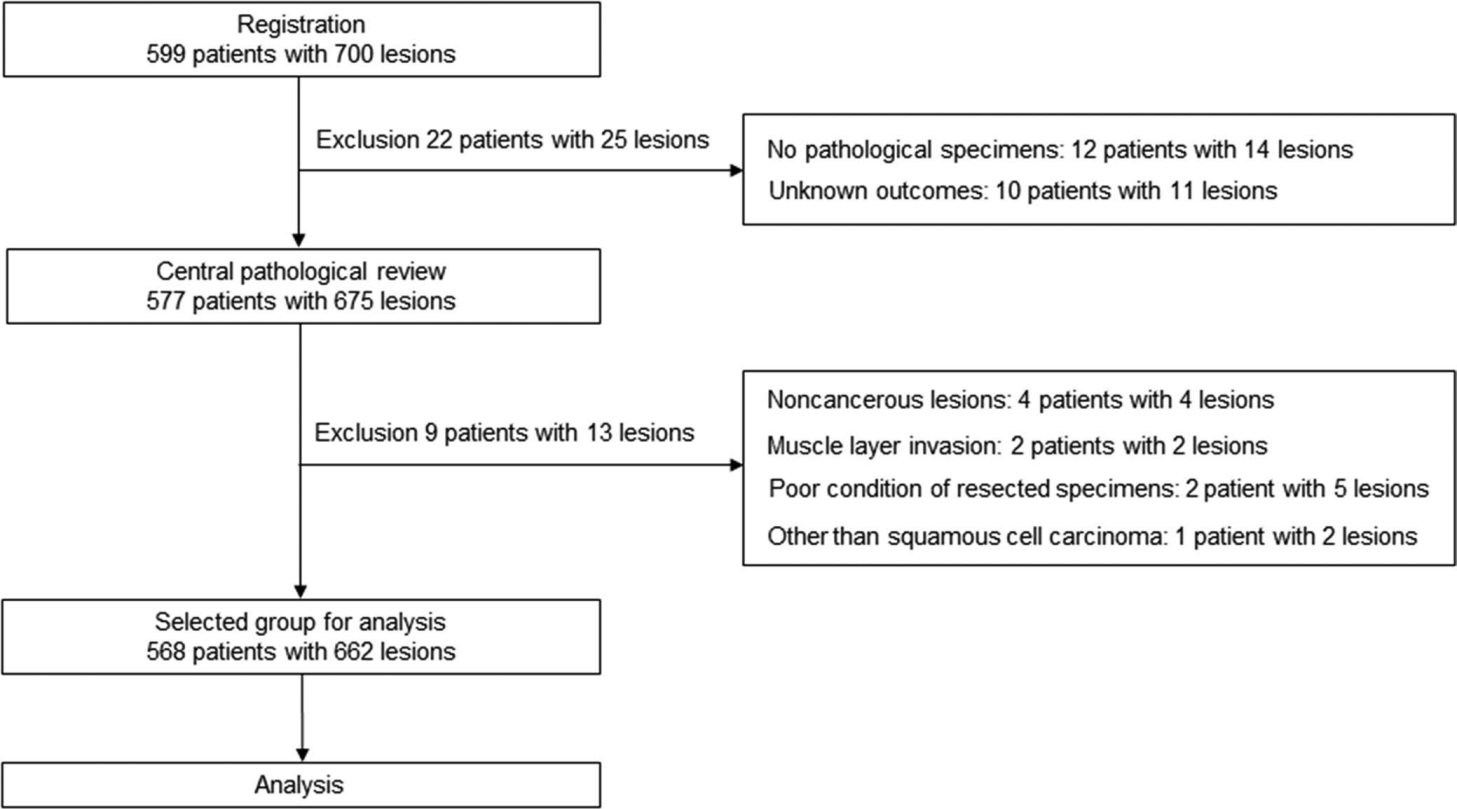
Flow chart of patients and lesions. ^*^Seven other sessions for synchronous lesions were performed on another day

A total of 599 patients with superficial head and neck cancer (700 lesions) were registered. We excluded 12 patients (14 lesions) with no available pathological specimens and 10 patients (11 lesions) with inadequate follow‐ups. The specimens of the remaining 577 patients (675 lesions) were carefully screened by one certified pathologist per protocol. This screening excluded 4 patients (4 lesions) with non‐cancerous lesions, 2 patients (2 lesions) with SCC invasion to muscular layer, 2 patients (5 lesions) with insufficient specimen to evaluate pathological findings, and 1 patient (2 lesions) with a tumor of a histologic type other than SCC (spindle cell carcinoma). Finally, a total of 568 patients (662 lesions) were included in the analysis. The total number of TOS sessions for 568 patients was 575 because 7 other sessions for synchronous lesions were performed on another day (Figure 1).

Table 1 shows the demographic characteristics of the study patients. The median age was 66 years (range 33 to 89), and 534 (94.0%) of the subjects were men. The performance status was 0 in 539 patients (94.9%). The most common reason for the detection of superficial head and neck cancer was endoscopic examination before or after the treatment of esophageal cancer (366 patients, 64.4%). The total number of patients with previous head and neck cancer and history of other cancer was 141 and 531 (includes overlapping patients), respectively. Among the 531 patients with previous cancer, 416 (78.3%) had history of esophageal cancer. Treatment for these cancers was summarized in Table 2.

**TABLE 1:** Patient characteristics

Total number of patients	568
Main factor leading to detection	
Before/after treatment of esophageal cancer	366 (64.4%)
Before/after treatment of head and neck cancer	83 (14.6%)
Medical checkups	55 (9.7%)
Pharyngolaryngeal paresthesia	49 (8.6%)
Before/after treatment of gastric cancer	15 (2.6%)
Age, median (range)	66 (33–89)
Sex (male)	534 (94.0%)
Performance status (0/1/2/3/4)	539 (94.9%) / 22 (3.9%) / 6 (1.1%) / 1 (0.2%) / 0 (0.0%)
History of head and neck cancer	
Total number of previous head and neck cancers^a^	141
Hypopharynx	43 (30.5%)
Oral cavity	38 (27.0%)
Larynx	31 (22.0%)
Oropharynx	25 (17.7%)
Primary unknown	2 (1.4%)
Maxilla	2 (1.4%)
History of cancer in other organ	
Total number of previous cancers^a^	531
Esophageal cancer	416 (78.3%)
Gastric cancer	74 (13.9%)
Colorectal cancer	12 (2.3%)
Prostate cancer	9 (1.7%)
Lung cancer	5 (0.9%)
Liver cancer	3 (0.6%)
Breast cancer	2 (0.4%)
Skin cancer	2 (0.4%)
Bladder cancer	2 (0.4%)
Malignant lymphoma	2 (0.4%)
Bile‐duct cancer	1 (0.2%)
Thyroid cancer	1 (0.2%)
Duodenal cancer	1 (0.2%)
Anal canal cancer	1 (0.2%)

^a^ Including overlapping patients.

**TABLE 2:** Treatment history of head and neck cancer and cancer in other organ

	Total number of previous cancers^a^	Surgery‐based therapy	Endoscopy‐based therapy	Chemoradiation –based therapy	Chemotherapy or hormonal therapy	Observation	TACE/RFA	Unknown
Head and neck cancer	141	75	0	60	5	1	0	0
Esophageal cancer	416	120	213	80	0	2	0	1
Gastric cancer	74	37	34	1	2	0	0	0
Colorectal cancer	12	9	2	0	1	0	0	0
Prostate cancer	9	4	0	1	4	0	0	0
Lung cancer	5	3	1	1	0	0	0	0
Liver cancer	3	1	0	0	0	0	2	0
Bladder cancer	2	1	0	1	0	0	0	0
Breast cancer	2	2	0	0	0	0	0	0
Skin cancer	2	1	1	0	0	0	0	0
Malignant lymphoma	2	1	0	1	0	0	0	0
Bile‐duct cancer	1	1	0	0	0	0	0	0
Duodenal cancer	1	1	0	0	0	0	0	0
Anal canal cancer	1	0	0	1	0	0	0	0
Thyroid cancer	1	1	0	0	0	0	0	0
Total	672	257	251	146	12	3	2	1

Abbreviations: RFA, radiofrequency ablation; TACE, transcatheter arterial chemoembolization.

^a^ Including overlapping patients.

Table 3 shows the demographic characteristics of the treated lesions. Among the 662 lesions, 519 (78.4%) were located in the hypopharynx and 132 lesions (19.9%) were located in the oropharynx. The most common macroscopic types was flat (528 lesions, 79.8%). The procedures for TOS were EMR (307 lesions, 46.2%), ESD (264 lesions, 39.7%), ELPS (31 lesions, 4.7%), and TOVS (31 lesions, 4.7%). A total of 490 lesions (74.0%) underwent *en bloc* resection. The median tumor diameter was 12 mm (range 1–75) endoscopically and 14 mm (range 1–60) pathologically. The median diameters of the resected tumor specimens in EMR, ESD, ELPS, TOVS, and other procedures were 12, 15, 20, 16, and 13 mm, respectively (ranges: 1–45, 1–60, 2–58, 5–42, and 3–50 mm, respectively). Three hundred and eighty lesions (57.4%) were revealed to be intraepithelial SCC based on the central pathological review on the depth of invasion. The T categories were found to be Tis (380 lesions, 57.4%), T1 (181 lesions, 27.3%), T2 (89 lesions, 13.4%), T3 (11 lesions, 1.7%), and unknown (1 lesion, 0.2%). Subsequent treatment was performed immediately after initial TOS for 20 lesions (3.0%).

**TABLE 3:** Lesion characteristics

Total number of lesions	662
Tumor location	
Oropharynx	132 (19.9%)
Anterior wall / Posterior wall / Lateral wall / Superior wall	9/79/23/21
Hypopharynx	519 (78.4%)
Postcricoid / Pyriform sinus / Posterior wall	33/404/82
Larynx	7 (1.1%)
Laryngeal epiglottis / Laryngeal arytenoid / Aryepiglottic folds	4/2/1
Oral cavity	4 (0.6%)
Oral floor / Hard palate / Buccal mucosa	1/1/2
Macroscopic type	
Flat / Elevated / Unknown	528 (79.8%) / 127 (19.2%) / 7 (1.1%)
Treatment methods	
Endoscopic mucosal resection (EMR)	307 (46.2%)
Endoscopic submucosal dissection (ESD)	264 (39.7%)
Endoscopic laryngopharyngeal surgery (ELPS)	31 (4.7%)
Transoral videolaryngoscopic surgery (TOVS)	31 (4.7%)
Laser microlaryngeal surgery	17 (2.6%)
Direct mucosectomy	12 (1.8%)
Number of resected specimens	
En bloc	490 (74.0%)
Piecemeal	172 (26.0%)
Number of segments obtained by piecemeal resection	
2/3/4/5/6/7/8/9/10/11	85/39/13/11/10/7/3/1/2/1
Tumor diameter on endoscopic images, median (range)^a^	12 (1–75)
Tumor diameter of resected specimens, median (range)^b^	14 (1–60)
EMR / ESD / ELPS / TOVS / Other procedures	12 (1–45) / 15 (1–60) / 20 (2–58) / 16 (5–42) / 13 (3–50)
Endoscopic depth of invasion for resected lesions	
Intraepithelial / Subepithelial / Difficult to evaluate	472 (71.0%) / 158 (23.8%) / 32 (4.8%)
Histopathological depth of invasion (central diagnosis)	
Intraepithelial / Subepithelial	380 (57.4%) / 282 (42.6%)
T category	
Tis / T1 / T2 / T3 / Unknown	380 (57.4%) / 181 (27.3%) / 89 (13.4%) / 11 (1.7%) / 1 (0.2%)
Lymphatic invasion	19 (2.9%)
Venous invasion	16 (2.4%)
Horizontal margin positive for cancer in the resected specimen	309 (46.7%)
Subsequent treatment immediately after initial transoral surgery	20 (3.0%)

^a^ Missing data for 29 patients.

^b^ Missing data for 1 patient.

### 3.2 Adverse events

Among the 575 treatment sessions, most of the procedure was underwent under general anesthesia (545 sessions, 94.8%). The median procedure time was 48 minutes (range 2–357). EMR was performed in a short time (32 minutes, *p *< 0.0001). Adverse events occurred in 12.7% (73/575). The main adverse events were laryngeal edema (33 sessions, 5.7%), subcutaneous emphysema (20 sessions, 3.5%), aspiration pneumonia (14 sessions, 2.4%), and bleeding (11 sessions, 1.9%). Subcutaneous emphysema frequently occurred in ESD (6.3%, *p *< 0.0178). Temporary tracheotomy was performed in 49 treatment sessions (8.5%). The main reasons for tracheotomy were development of laryngeal edema (22 sessions, 3.8%) and perioperative planned management (21 sessions, 3.7%). There were no treatment‐related deaths; however, 3 patients (0.5%) developed life‐threatening severe adverse events. Those were as follows: 1) one patient underwent an emergency tracheotomy because of suffocation caused by laryngeal edema after surgery, 2) one patient underwent an emergency tracheotomy because of arterial bleeding and hemostasis was achieved by ligation of the blood vessels, and 3) one patient had transient cardiopulmonary arrest caused by aspiration of food during a meal on the following day and recovered after removal of the foreign object through a tracheostoma (Table 4).

**TABLE 4:** Variables related to the safety of transoral surgery

	Total	Endoscopic mucosal resection	Endoscopic submucosal dissection	Other procedures	*p*‐value
Total number of treatment sessions	575	263	222	90	
Methods for anesthesia					0.0353
General anesthesia	545 (94.8%)	242 (92.0%)	214 (96.4%)	89 (98.9%)	
Intravenous anesthesia	29 (5.0%)	20 (7.6%)	8 (3.6%)	1 (1.1%)	
None	1 (0.2%)	1 (0.4%)	0 (0.0%)	0 (0.0%)	
Procedure time, median (range), min^a^	48 (2–357)	32 (2–240)	60 (15–357)	71 (6–300)	<0.0001
Adverse events	73 (12.7%)	28 (10.6%)	36 (16.2%)	9 (10.0%)	0.1399
Laryngeal edema	33 (5.7%)	18 (6.8%)	13 (5.9%)	2 (2.2%)	0.2593
Subcutaneous emphysema	20 (3.5%)	5 (1.9%)	14 (6.3%)	1 (1.1%)	0.0178
Aspiration pneumonia	14 (2.4%)	4 (1.5%)	9 (4.1%)	1 (1.1%)	0.1704
Bleeding	11 (1.9%)	5 (1.9%)	5 (2.3%)	1 (1.1%)	0.923
Stenosis	3 (0.5%)	1 (0.4%)	2 (0.9%)	0 (0.0%)	0.7573
Cerebral infarction	2 (0.3%)	1 (0.4%)	1 (0.5%)	0 (0.0%)	1
Dermatitis caused by iodine	1 (0.2%)	1 (0.4%)	0 (0.0%)	0 (0.0%)	1
Tooth injury	1 (0.2%)	0 (0.0%)	0 (0.0%)	1 (1.1%)	0.1565
Mediastinitis	1 (0.2%)	0 (0.0%)	1 (0.5%)	0 (0.0%)	0.5426
Temporary tracheotomy	49 (8.5%)	22 (8.4%)	22 (9.9%)	5 (5.6%)	0.4964
Reason for tracheotomy					
Development of laryngeal edema^b^	22 (3.8%)	17 (6.5%)^b^	4 (1.8%)	1 (1.1%)	0.0113
Perioperative planned management^b^	21 (3.7%)	6 (2.3%)^b^	12 (5.4%)	3 (3.3%)	0.1869
Difficulty for intraoperative bleeding management	2 (0.3%)	0 (0.0%)	1 (0.5%)	1 (1.1%)	0.1453
Unknown	5 (0.9%)	0 (0.0%)	5 (2.3%)	0 (0.0%)	0.0243
Life‐threatening severe adverse event	3 (0.5%)	1 (0.4%)	2 (0.9%)	0 (0.0%)	0.7573
Treatment‐related death	0 (0%)	0 (0%)	0 (0%)	0 (0%)	—

^a^ Missing data for 21 patients.

^b^ One overlapping patient.

### 3.3 Local, regional lymph node and distant recurrence

Median follow‐up period was 46.1 months (range 1–113). Recurrence data and their treatment were summarized in Table 5. Among 662 lesions treated by TOS, 53 lesions (8.0%) developed local recurrence. Local recurrence rates of EMR, ESD, and other procedures were 11.7% (35/298), 2.7% (7/258), and 10.4% (11/106), respectively (*p *< 0.0001). The median diameters of the resected tumor specimens which developed local recurrence and specimens that did not develop local recurrence were 16 (range: 3–45 mm) and 14 mm (range: 1–60 mm), respectively. There was no relation between tumor size and local recurrence (*p *= 0.13). Thirty‐nine lesions (73.6%) were treated by re‐TOS. Traditional open surgery with and without laryngectomy were performed in 3 lesions (5.7%) and 2 lesions (3.8%), respectively. Remaining 9 lesions (17.0%) were treated with non‐surgical treatment. Regional lymph node recurrence developed in 26 patients (4.6%). Among them, 20 patients (76.9%) developed on the same side of the neck. Radical neck dissection was performed in 15 patients (57.7%), and 8 patients (30.8%) received neck dissection plus postoperative chemotherapy and/or radiotherapy. Three patients (11.5%) received definitive chemoradiotherapy. Three patients (0.5%) had distant recurrence; two had lung metastasis and remaining one had lung and liver metastasis. Two patients (66.7%) were followed up without any treatment, and 1 patient (33.3%) received chemotherapy.

**TABLE 5:** Recurrence, metachronous head and neck cancer, and their treatment after transoral surgery

Local recurrence (n = 662 lesions)	53 (8.0%)
Treatment for recurrent lesions	
Transoral surgery	39 (73.6%)
Traditional open surgery	5 (9.4%)
With laryngectomy	3 (5.7%)
Without laryngectomy	2 (3.8%)
Observation	3 (5.7%)
Definitive chemoradiotherapy	2 (3.8%)
Radiotherapy	2 (3.8%)
Argon plasma coagulation	1 (1.9%)
Laser ablation	1 (1.9%)
Regional lymph node recurrence (n = 568 patients)	26 (4.6%)
Location of recurrent lesions	
Only same side	20 (76.9%)
Only opposite side	2 (7.7%)
Both sides	2 (7.7%)
Unknown	2 (7.7%)
Treatment for recurrent lesions	
Neck dissection	15 (57.7%)
Neck dissection + postoperative chemotherapy	3 (11.5%)
Neck dissection + postoperative radiotherapy	3 (11.5%)
Neck dissection + postoperative chemoradiotherapy	2 (7.7%)
Definitive chemoradiotherapy	3 (11.5%)
Distant recurrence (n = 568 patients)	3 (0.5%)
Location of recurrent lesions	
Lung	2 (66.7%)
Lung + Liver	1 (33.3%)
Treatment for recurrent lesions	
Chemotherapy	1 (33.3%)
Observation	2 (66.7%)
Metachronous head and neck cancer (n = 568 patients)	132 (23.2%) with 234 lesions
Treatment for metachronous lesions	
Transoral surgery	207 (88.5%)
Traditional open surgery	5 (2.1%)
With laryngectomy	1 (0.4%)
Without laryngectomy	4 (1.7%)
Argon plasma coagulation	9 (3.8%)
Radiotherapy	6 (2.6%)
Observation	3 (1.3%)
Definitive chemoradiotherapy	1 (0.4%)
Chemotherapy	1 (0.4%)
Unknown	2 (0.9%)

### 3.4 Metachronous cancer

**FIGURE 2:**
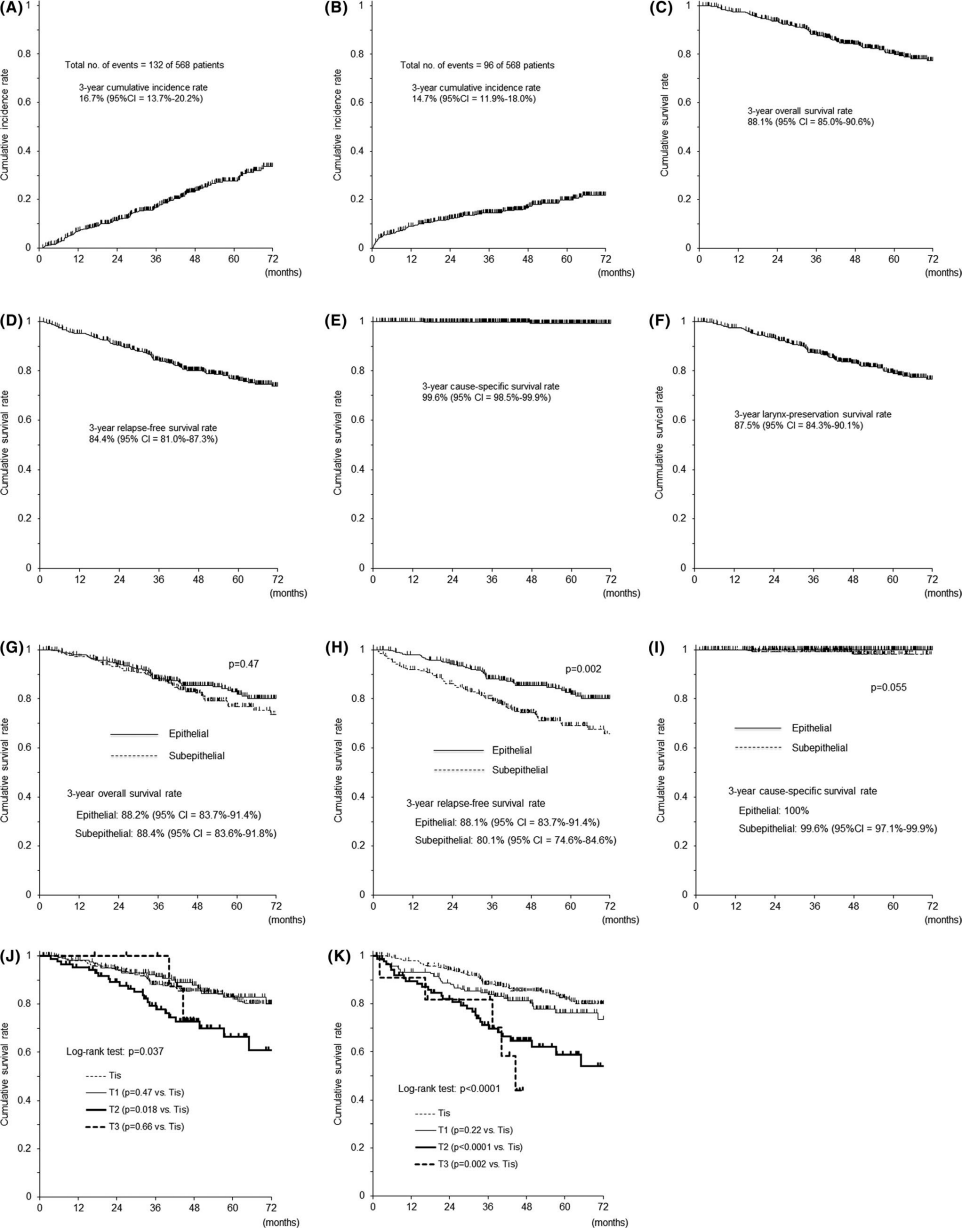
Cumulative incidence of metachronous cancers and survival rate. A, Cumulative incidence rate of metachronous head and neck cancers. B, Cumulative incidence rate of metachronous cancers arising in other organs. C, Overall survival rate. D, Relapse‐free survival rate. E, Cause‐specific survival rate. F, Larynx‐preservation survival rate. G, Overall survival rates according to the histopathological depth of invasion. H, Relapse‐free survival rates according to the histopathological depth of invasion. I, Cause‐specific survival rates according to the histopathological depth of invasion. J, Overall survival rates according to the T category. K, Relapse‐free survival rates according to the T category

A total of 234 metachronous head and neck cancers were diagnosed in 132 patients (23.2%) during the follow‐up period. The 3‐year cumulative incidence rate of metachronous head and neck cancers after TOS was 16.7% (95% confidence interval, 13.7% to 20.2%) (Figure 2A). Among 234 lesions, 207 (88.5%) were again treated by TOS. Traditional open surgery with and without laryngectomy were performed in 1 lesion (0.4%) and 4 lesions (1.7%), respectively. Other 20 lesions (8.5%) were treated with non‐surgical treatment and the treatment details of 2 lesions (0.9%) were not available (Table 5).

A total of 131 metachronous cancers arising in other organ were diagnosed in 96 patients (16.9%) during the follow‐up period. The 3‐year cumulative incidence rate of metachronous cancers arising in other organs after TOS was 14.7% (95% CI, 11.9% to 18.0%) (Figure 2B). And treatment for these cancers were summarized in Table 6. Esophagus was the main sites of metachronous cancer arising in other organ. Among 90 metachronous esophageal cancers, endoscopic resection was performed in 74 lesions (82.2%), surgery‐based treatment in 11 lesions (12.2%), and chemoradiation‐based treatment in 5 lesions (5.6%).

**TABLE 6:** Treatment of metachronous cancer arising in other organ

	Total number of metachronous cancers^a^	Endoscopic resection	Surgery‐based therapy	Chemoradiation‐based therapy	Chemotherapy or hormonal therapy	Observation	TACE/RFA^b^
Esophageal cancer	90	74	11	5	0	0	0
Gastric cancer	16	12	4	0	0	0	0
Lung cancer	10	1	6	2	1	0	0
Colorectal cancer	5	1	3	0	0	1	0
Bile‐duct cancer	4	0	3	0	1	0	0
Liver cancer	2	0	1	0	0	0	1
Duodenal cancer	1	0	0	0	0	1	0
Prostate cancer	1	0	0	0	1	0	0
Urinary tract cancer	1	0	1	0	0	0	0
Thyroid cancer	1	0	1	0	0	0	0
Total	131	88	30	7	3	2	1

^a^ Including overlapping patients.

^b^ TACE: transcatheter arterial chemoembolization, RFA: radiofrequency ablation.

### 3.5 Survival

During a median follow‐up period of 46.1 months (range 1–113), 3 patients died of superficial head and neck cancer because of 2 distant metastasis and 1 local lymph node metastasis and 25 patients died of metachronous cancer arising in other organ. Specific sites of cancer among those 25 patients were esophageal cancer in 8 patients, lung cancer in 6 patients, colorectal cancer in 3 patients, gastric cancer in 2 patients, liver cancer in 2 patients, bile duct cancer in 2 patients, duodenal cancer in 1 patient, and ureteral cancer in 1 patient.

The 3‐year overall survival rate (Figure 2C) was 88.1% (95% CI, 85.0% to 90.6%), the 3‐year relapse‐free survival rate (Figure 2D) was 84.4% (95% CI, 81.0% to 87.3%), the 3‐year cause‐specific survival rate (Figure 2E) was 99.6% (95% CI, 98.5% to 99.9%), and the 3‐year larynx‐preservation survival rate (Figure 2F) was 87.5% (95% CI, 84.3% to 90.1%).

Survival was analyzed based on the depth of invasion (carcinoma in situ vs. cancer with subepithelial invasion) and results were compared. The 3‐year overall survival rates (Figure 2G), the 3‐year relapse‐free survival rates (Figure 2H), and the 3‐year cause‐specific survival rates (Figure 2I) were 88.2% (95% CI, 83.7% to 91.4%) vs. 88.4% (95% CI, 83.6% to 91.8%) (*p *= 0.47), 88.1% (95% CI, 83.7% to 91.4%) vs. 80.1% (95% CI, 74.6% to 84.6%) (*p *= 0.002), and 100% vs. 99.6% (95% CI, 97.1% to 99.9%) (*p *= 0.055), respectively.

Survival based on the T category was analyzed. The 3‐year overall survival rates of Tis, T1, T2, and T3 tumors (Figure 2J) were 88.2% (95% CI, 83.7% to 91.4%), 92.2% (95% CI, 86.7% to 95.5%), 79.3% (95% CI, 68.4% to 86.8%), and 100% (*p *= 0.037), respectively. The 3‐year relapse‐free survival rates of Tis, T1, T2, and T3 tumors (Figure 2K) were 88.1% (95% CI, 83.7% to 91.4%), 84.7% (95% CI, 78.0% to 89.5%), 71.2% (95% CI, 59.8% to 79.9%), and 81.8% (95% CI, 44.7% to 95.1%) (*p *< 0.0001), respectively. The 3‐year cause‐specific survival rates of Tis, T1, T2, and T3 tumors were 100%, 98.7% (95% CI, 94.9% to 99.7%), 100%, and 100% (*p *= 0.068), respectively.

## 4 DISCUSSION

This is the first report of national multi‐center survey of TOS for superficial head and neck cancer based on the standardized pathological evaluation. During a median follow‐up period of 46.1 months (range 1–113), the 3‐year overall survival rate and the 3‐year cause‐specific survival rate was 88.1% (95% CI. 85.0% to 90.6%) and 99.6% (95% CI, 98.5% to 99.9%), respectively. There was no treatment‐related death.

The most important clinical benefit of TOS was that it could preserve organ and function sparing patients from potentially devastating adverse events of radial surgery or chemoradiation. In this study, a total of 53 local recurrence (8.0%) developed after completion of TOS. However, 39 recurrent lesions (73.6%) were treated by re‐TOS. As for regional lymph node recurrence, most of the patients were treated by radical neck dissection or radical neck dissection plus chemotherapy and/or radiotherapy. Only 4 patients (0.7%) underwent laryngectomy (3 for local recurrence and 1 for metachronous head and neck cancer). Therefore, 99.3% (564/568) of the patients overall enjoyed preservation of organ and function. Calculated 3‐year larynx‐preservation survival rate was very high at 87.5%.

The local recurrence rate was significantly lower in the *en bloc* resection (5.3%) than in the piecemeal resection (15.7%, *p *< 0.0001). Previous studies reported that the size of tumors that can be resected *en bloc* by EMR is limited and that EMR tends to have a higher rate of local recurrence.^30^, ^31^ In this study, *en bloc* resection rate of EMR was 55.4% for median tumor size of 14 mm. Because the average size of *en bloc* resected specimens by EMR is 10.3 ± 6.1 mm, EMR may be suitable for small lesions if *en bloc* resection can be performed.

The most frequent adverse event was laryngeal edema. Temporary tracheostomy was indicated in 49 (8.5%) of 575 treatment sessions. Among them, 22 procedures (44.9%, 22/49) directly attributed to laryngeal edema and 2 procedures (0.3%) were due to difficulty for intraoperative bleeding management. In contrast, 21 procedures (42.9%, 21/49) were indicated for the planned tracheostomy to avoid airway obstruction potentially caused by laryngeal edema, bleeding, or aspiration after TOS even in the cases with absence of intraoperative adverse events. However, the indication for planned tracheostomy was not clear because all such adverse events did not cause airway obstruction. Then, we have to clear the definite indication of planned tracheostomy to introduce the TOS as a minimally invasive treatment.

The rate of postoperative stenosis in the present study was only 0.5% (3/575). In the three cases who developed stenosis, the pathological tumor diameters were 15, 16, and 45 mm, respectively. And, all lesion located in the pyriform sinus. The possible reason developed stenosis might be associated with the tumor lesion regardless of the tumor size because the pyriform sinus is directly connected to the cervical esophagus which is physiological stenotic part.

Indication for TOS has not been clearly determined. In this study, pathological criteria for intraepithelial SCC and subepithelial SCC have been clearly defined. Using this criteria, relapse‐free survival rates were significantly different between two groups, while overall survival was similar. Cause‐specific survival rate was not statistically different between the two groups because both groups had nearly 100% cause‐specific survival. These results indicated that our pathological criteria for subepithelial invasion is clinically useful to stratify the risk for recurrence but not survival after TOS.

Early detection of head and neck cancer continues to be difficult worldwide. Screening of cancer in the head and neck is not a common practice. However, early detection is important because advanced head and neck cancer has poor prognosis and conventional treatments adversely affect the patients’ quality of life. Image enhanced endoscopy such as NBI is revealed to be useful for early detection of head and neck cancer.^12^ However, it is not routinely used in Western countries, while they were high incidence area for head and neck cancer. We would like to emphasize the benefit of image‐enhanced endoscopy and hope it will be used in routine clinical practice especially in countries with known high incidence of head and neck cancer.

Our study has several limitations. This is a retrospective study and the duration of follow‐up was relatively short. Although this national multi‐center survey showed real‐world outcomes and benefit of TOS for superficial head and neck cancer and we have shown clinically meaningful pathological criteria of subepithelial invasion, a prospective study would provide a better assessment of individual management of TOS.

In conclusion, TOS for superficial head and neck cancer appears to be an excellent organ preserving minimally invasive treatment that results in excellent cause‐specific survival.

## CONFLICT OF INTEREST

Professor Manabu Muto received a joint research fund between Kyoto University and Olympus Corporation out of this study. Dr. Ryuichi Hayashi received a grant from the Japan Agency for Medical Research and Development (Grant No. 19ck0106510h0001). Other authors have not declared a specific grant for this study from any funding agency in the public, commercial, or not‐for‐profit sectors.

## AUTHOR CONTRIBUTIONS

CK, MM, SF, TEY, YAS, HW, TS, AO, TAK, and RH contributed to conception and design. CK, KOK, YAS, and MI contributed to collection and assembly of data. CK, MM, SF, TEY, YAS, and RH contributed to data analysis and interpretation. CK, MM, SF, and TEY wrote the manuscript. RH provided financial support. CK, KOK, YAS, MI, MM, and RH contributed to administrative support. TOY, AW, TI, SY, IT, HM, YUS, AT, TOK, NH, MA, AS, KOK, YA, TM, HU, KO, KG, SH, YO, ST, YN, KT, KEK, MA, NS, and AA provided study materials or patients. All authors provided final approval of manuscript.

## ETHICAL CONSIDERATION

This study was approved by the institutional review board at Kitasato University School of Medicine (Approval ID; B10‐134).

## Data Availability

The data and other items supporting the results of the study will be made available upon reasonable request.
